# Upregulated NNT-AS1, a long noncoding RNA, contributes to proliferation and migration of colorectal cancer cells in vitro and in vivo

**DOI:** 10.18632/oncotarget.13840

**Published:** 2016-12-09

**Authors:** Qian Wang, Lei Yang, Xin Hu, Yuliang Jiang, Yizhang Hu, Zhe Liu, Jian Liu, Tao Wen, Yingmin Ma, Guangyu An, Guosheng Feng

**Affiliations:** ^1^ Department of Oncology, Affiliated Beijing Chaoyang Hospital, Capital Medical University, Beijing 100020, China; ^2^ Department of Respiratory Medicine, Affiliated Beijing Chaoyang Hospital, Capital Medical University, Beijing 100020, China; ^3^ Medical Research Center, Affiliated Beijing Chaoyang Hospital, Capital Medical University, Beijing 100020, China

**Keywords:** long non-coding RNA, NNT-AS1, colorectal cancer, MAPK/Erk, biomarker

## Abstract

The expression patterns of the long non-coding RNA Nicotinamide Nucleotide Transhydrogenase-antisense RNA1 (NNT-AS1) have not been investigated in the context of cancer. In this study, we aim to investigate the NNT-AS1 expression level in colorectal cancer (CRC) patients and its potential roles in tumor biology. We measured the expression of NNT-AS1 in 70 paired tumor tissues and adjacent normal tissues. NNT-AS1 was expressed higher in tumor tissues than that in adjacent noncancer tissues, and higher expression of NNT-AS1 was significantly correlated with lymph node metastasis (Yes vs. No, P=0.004), TNM stage (I/II vs. III/IV, P=0.004), vessel invasion (Yes vs. No, P=0.002) and differentiation (well and moderate vs. poor, P=0.008). Multivariate analyses revealed that NNT-AS1 expression was an independent predictor of overall survival (P=0.0174) and progression free survival (P=0.0132) for CRC. Knockdown of NNT-AS1 using small interfering RNA (siRNA) significantly impaired CRC cell proliferation, migration and invasion in vitro and silencing NNT-AS1 also suppressed tumor growth and metastasis in nude mice. The western blot experiments revealed that silencing NNT-AS1 inhibited epithelial-mesenchymal transition (EMT) and inactivated MAPK/Erk signaling pathway in CRC cell lines. In conclusion, our studies implied that NNT-AS1 may involve in the development and progression of CRC via its regulation of cell proliferation, migration, and invasion by NNT-AS1-mediated activating of MAPK/Erk signaling pathway and EMT. NNT-AS1 may be a useful diagnostic and prognostic biomarker and a potential therapeutic target in CRC patients.

## INTRODUCTION

Colorectal cancer (CRC) is the third most common cancer and the third leading cause of cancer-related death in the world. There are approximately 1.4 million CRC cases and 693,900 deaths every year [1]. The overall 5-year survival rate is 64.9% in colon cancer and 66.5% in rectal cancer. Approximately 25% patients have metastasis at the time of diagnosis, which leads to the poor treatment outcome and poor prognosis of these patients [2]. Therefore, a biomarker with high specificity and sensitivity is desperately needed for early diagnosis, which can help to improve the curative effect and give insight into the pathogenesis of CRC.

Long noncoding RNAs (LncRNAs) are over 200 nucleotides in length without protein-coding capacity [3]. Abundant evidence demonstrates that LncRNAs are of functional importance. LncRNAs can mediate genes activation and inactivation by chromatin remodeling, such as Hox transcript antisense intergenic RNA (HOTAIR), X inactive specific transcript (XIST). They can regulate the functions of cells, such as differentiation, apoptosis and cell cycle by transferring the chromatin-modifying complexes to the promoters of important genes [4, 5]. They also can participate in transcriptional and post-transcriptional processing and protein modulating by binding to proteins [6], inhibiting promoters [7], interacting with transcription factors [8], or acting as endogenous sponges to sequester microRNAs [9]. With the above molecular mechanisms, lncRNAs can affect the development, progression and metastasis of cancers.

Recently, an increasing number of studies demonstrated that the upregulated lncRNA could be a useful biomarker in cancers [10]. HOTAIR has proved overexpressed in breast carcinoma, and its higher expression in primary tumors can predict a poor prognosis [11]. Colon cancer associated transcript 2 (CCAT2), higher expressed in cancer tissues than that in adjacent mucosa, can also be a diagnostic and prognostic biomarker of CRC [12].

**Figure 1:**
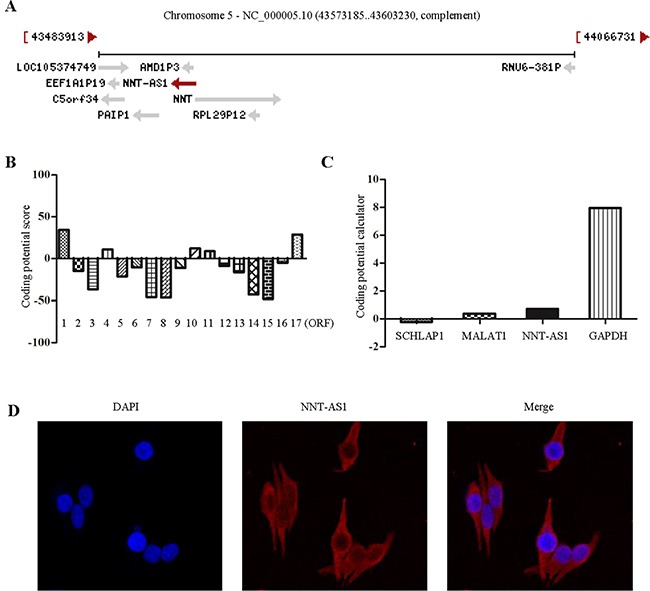
NNT-AS1 most likely has no protein coding potential **A**. NNT-AS1 is mapped to chromosome 5. **B**. Analyze protein coding potential of 17 ORFs of NNT-AS1 using PhlyoCSF. **C**. Analyze protein coding potential of NNT-AS1 and other genes using coding potential calculator (CPC). **D**. NNT-AS1 is mainly located in the cytoplasm by fluorescence in situ hybridization (FISH).

NNT-AS1, which located in 5p12 with 3 exons, has been mapped to chromosome 5 region 43573185-43603230 according to the NCBI (GRCh38.p2) (Figure 1A). NNT-AS1 is transcribed in the opposite direction of nicotinamide nucleotide transhydrogenase (NNT), but there is no overlap between NNT-AS1 and NNT. It is a newly detected lncRNA, and its role in cancers remains largely unknown. In our previous study, we gathered four datasets in CRC, including E-GEOD-31737, E-MATB-829, Affymetrix colon cancer dataset, and E-GEOD-24550, and we found that NNT-AS1 was highly expressed in CRC cancer tissues comparing to adjacent noncancer tissues (data has not been published).

Therefore, in the current study, our aim is to go deeper into NNT-AS1, to assess whether this lncRNA can be a useful diagnostic and prognostic biomarker, as well as a potential therapeutic target for CRC patients. We measured the expression level of NNT-AS1 in CRC cancer tissues and adjacent non-cancer tissues, and we also assessed the roles of NNT-AS1 in CRC tumor biology, and the underlying mechanism using CRC cell models.

## RESULTS

### NNT-AS1 has no protein coding potential

We analyzed the coding potential in 17 Open Reading Frames (ORFs) of NNT-AS1 with the PhlyoCSF [13]. The results showed that most of the ORFs (12/17) had no protein coding potential (Figure 1B). A score of -10 means that the non-coding potential is 10 times more than coding potential, while a score of 10 indicates that the coding potential is 10 times more than noncoding potential. In addition, we also used Coding Potential Calculator (CPC) to measure the coding potential of NNT-AS1 and some other non-coding RNAs and coding RNA (Figure 1C), We also found that NNT-AS1 have significantly lower score comparing to the coding RNA. As expected, NNT-AS1 is mainly located in the cytoplasm by fluorescence in situ hybridization (FISH) assays in SW620 cell lines (Figure 1D).

### NNT-AS1 expression was increased in CRC tissues

**Figure 2:**
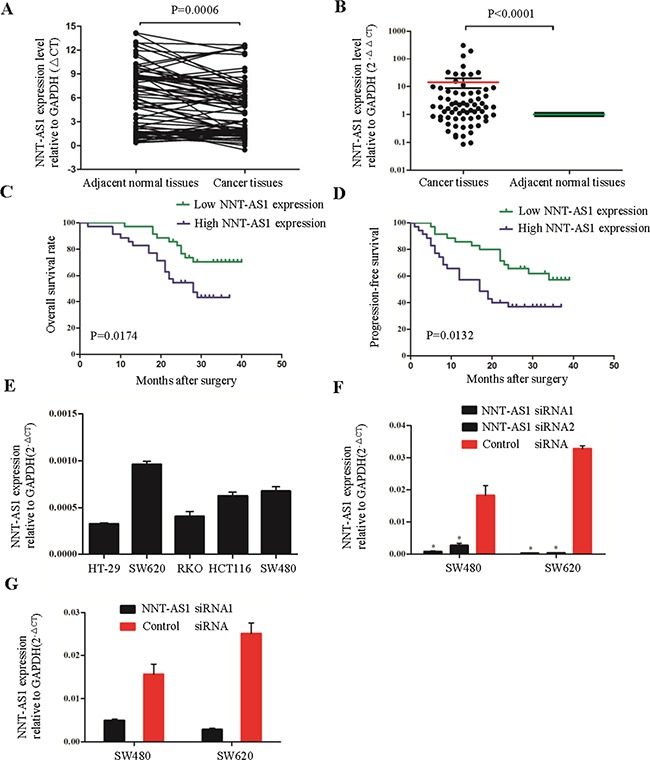
NNT-AS1 is overexpressed and associated with survival of CRC patients **A**. and **B**. Quantitative RT-PCR analysis of NNT-AS1 expression levels in 70 CRC patients. The results were expressed as ΔCt (A) and 2^-ΔΔCt^ (B). **C**. and **D**. Kaplan-Meier analysis of OS (C) and PFS (D) in 70 CRC patients. Patients are divided into NNT-AS1 high expression group (ΔCt ≤4.25) and low expression group(ΔCt > 4.25) according to the median level of NNT-AS1 in cancer tissues. **E**. The expression levels of NNT-AS1 in 5 CRC cell lines. **F**. NNT-AS1 expression after transfecting NNT-AS1-specific siRNA1 and siRNA2 were confirmed by qRT-PCR in SW480 and SW620. **G**. The knock-down efficiency of stable cells after transfecting vectors were measured in SW620 (*P<0.01).

NNT-AS1 expression was examined in cancerous tissues and adjacent noncancerous tissues in 70 CRC patients by quantitative real-time PCR (qRT-PCR). Significantly higher NNT-AS1 expression was found in the cancerous tissues than that in the adjacent normal tissues (Figure 2A and 2B). When the 70 tumor tissue samples were stratified into higher and lower expression groups using the median expression levels as a cutoff (ΔCT=4.25), we found that higher NNT-AS1 expression (ΔCT≤4.25) was significantly correlated with lymph node metastasis (Yes vs. No, P=0.004), TNM stage (I/II vs. III/IV, P=0.004), vessel invasion (Yes vs. No, P=0.002) and tumor differentiation (well and moderate vs. poor, P=0.008) (Table 1).

**Table 1:** Relationship between NNT-AS1 expression and clinicopathological characteristics in colorectal cancer patients

Characteristics	Number	NNT-AS1 expression		P value
		High(n=35)	Low(n=35)	
Age(years)				
<60	20(29%)	12	8	0.306
≥60	50(71%)	23	27	
Gender				
Male	47(67%)	25	22	0.214
Female	23(33%)	10	13	
TNM stage				
I, II	36(51%)	12	24	0.004
III, IV	34(49%)	23	11	
Differentiation				
Well, moderate	50(71%)	20	30	0.008
Poor	20(29%)	15	5	
Vessel invasion				
Yes	33(47%)	23	10	0.002
No	37(53%)	12	25	
Neural invasion				
Yes	23(33%)	14	9	0.154
N0	47(67%)	21	26	
Tumor invasion depth				
T1, T2	8(11%)	2	6	0.130
T3, T4	62(89%)	33	29	
Lymph node metastasis				
Yes	34(49%)	23	11	0.004
No	36(51%)	12	24	
Distant metastasis				
M1	1(1%)	1	0	0.500
M0	69(99%)	34	35	

### Univariate and multivariate analyses indicated that NNT-AS1 expression was an independent predictor of overall survival and progression-free survival

Kaplan-Meier analysis and log-rank tests were used to further evaluate the correlation between NNT-AS1 expression and overall survival (OS) of CRC patients. We found that OS / progression-free survival (PFS) were both significantly shorter in the patients with higher NNT-AS1 expression (P=0.0174 and P=0.0132 respectively) (Figure 2C and 2D).

By univariate analysis, three prognostic factors for OS were identified: vessel invasion (Yes vs. No, P=0.004), TNM stage (I/II vs. III/V, P=0.030), and NNT-AS1 expression level (Higher vs. Lower, P=0.023). Furthermore, multivariate analysis revealed that NNT-AS1 expression was a significant independent predictor of poor survival in CRC patients (P=0.024) as well as tumor vessel invasion (P=0.004) (Table 2).

**Table 2:** Univariate and multivariate Cox regression analysis of overall survival in 70 colorectal cancer patients

Variables	Univariate analysis		Multivariate analysis	
	HR (95% CI)	P value	HR (95% CI)	P value
Age	1.004(0.972-1.036)	0.825		
Gender	0.854 (0.389-1.876)	0.694		
Differentiation	0.496(0.237-1.040)	0.063		
Neural invasion	1.787 (0.858-3.723)	0.121		
TNM stage	**0.426 (0.198-0.920)**	0.030*	1.678 (0.750-3.752)	0.208
Lymph node metastasis	**2.345(1.087-5.057)**	0.030*		
Vessel invasion	**3.040 (1.432-6.457)**	0.004*	**3.034 (1.427-6.453)**	0.004*
NNT-AS1 expression	**2.439(1.132-5.255)**	0.023*	**2.431 (1.127-5.244)**	0.024*

* Statistically significant difference; HR, hazard ratio; CI, confidence interval.

### The knock-down efficiency of two siRNA molecules in CRC cells

To assess the functional roles of NNT-AS1 in CRC development and progression, NNT-AS1 expression was measured in 5 CRC cell lines (Figure 2E). Two cell lines (SW480 and SW620) which expressed higher NNT-AS1 were transfected with two NNT-AS1-specific siRNA and control siRNA. Seventy-two hours after transfection, qRT-PCR revealed that NNT-AS1 expression was obviously downregulated in both cell lines (Figure 2F). Of the two siRNAs, NNT-AS1 siRNA1 (s501613) demonstrated more robust silencing capacity both in SW480 and SW620 (Figure 2G). Thus, s501613 siRNA was cloned into a vector to do the experiments in colony formation assays and in nude mice assays below.

### Silencing NNT-AS1 inhibits cell proliferation, colony formation and cell cycle in vitro

**Figure 3:**
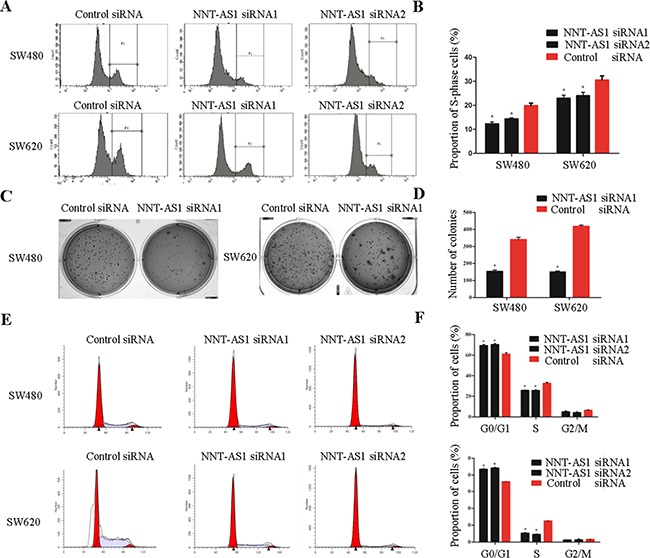
Function analysis of NNT-AS1 in vitro **A**. and **B**. Representative images of EdU assays in CRC cells. The percentage of proliferation cells is shown in the right panel (*P<0.01). **C**. and **D**. Representative images of colony assays and the number of colonies is shown in the right panel (*P<0.01). **E**. and **F**. Representative images of cell cycle assays and the percentage of each phase is shown in the right panel (*P<0.01). Date were presented as mean±SD.

To elucidate the function of NNT-AS1 in CRC, we subjected CRC cells to EdU assays and colony formation assays. The results of the EdU assays showed that the percentages of proliferating cells significantly decreased in both cell lines after silencing NNT-AS1 comparing to control group (Figure 3A and 3B). The colony formation assays showed that NNT-AS1 knockdown resulted in fewer colonies than that in control groups in both cell lines (P<0.05) (Figure 3C and 3D).

The cell cycle assays showed that the cells transfected with NNT-AS1-specific RNAs have significantly fewer cells in S-phase and notably stalled cells in G1-G0 phase comparing to that in control group (Figure 3E and 3F). However, in cell apoptosis assays, no significant differences were found between the cells transfected with NNT-AS1-specific RNAs and control siRNA (data not shown).

### NNT-AS1 knockdown inhibits CRC cell migration and invasion in vitro

**Figure 4:**
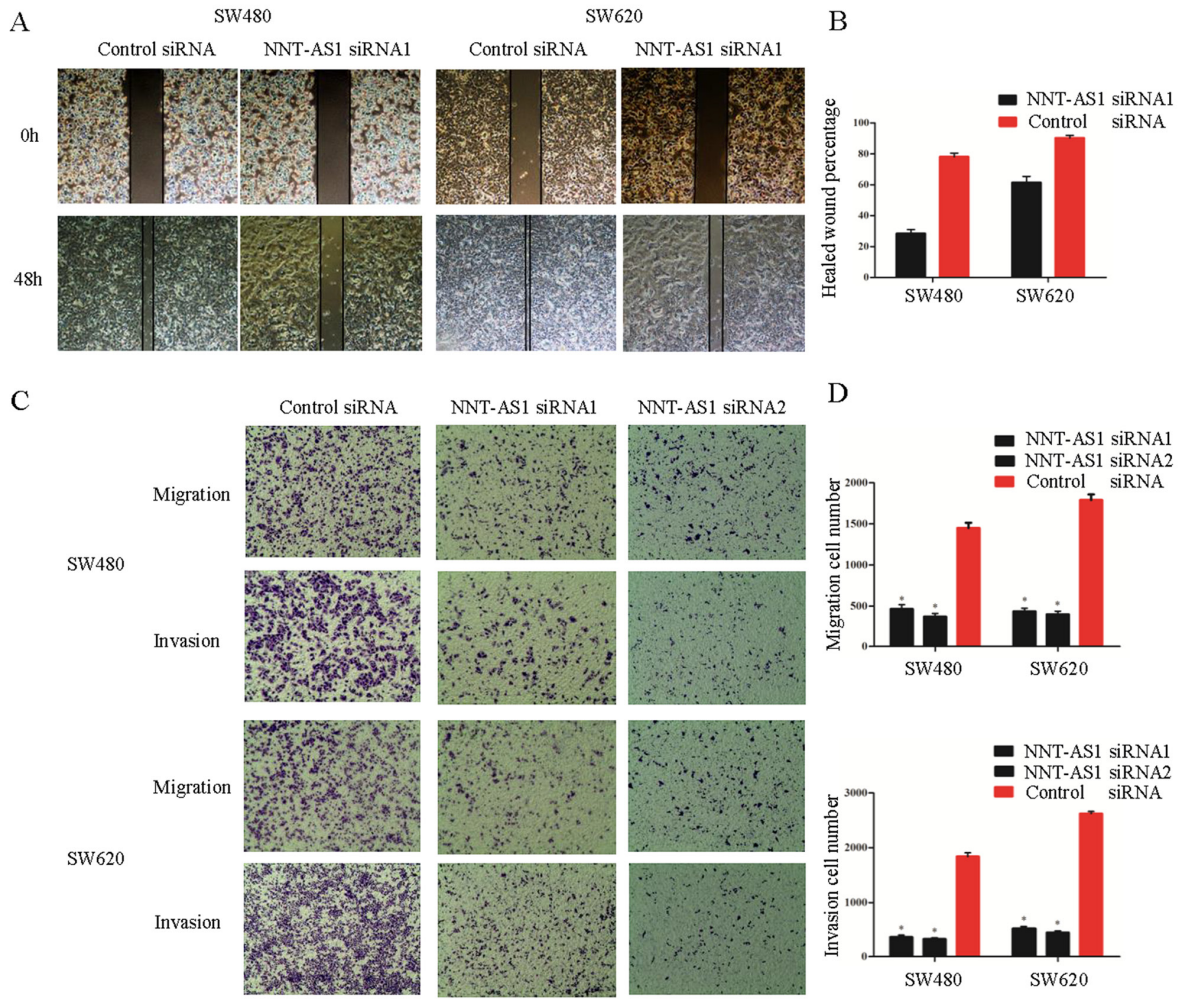
NNT-AS1 promotes CRC cell migration and invasion in vitro **A**. and **B**. Wound healing assays and would closure percentages are shown in the right panel (*P<0.01). **C**. and **D**. Cell migration assays after 24h and cell invasion assays after 50h. Quantitative results are shown in the right panel (*P<0.01). Date were presented as mean±SD.

To assess how NNT-AS1 affects the metastatic abilities in CRC cells, the wound healing assays and transwell assays were employed using the transfected cells. The wound healing assay showed that silencing NNT-AS1 significantly attenuated CRC migration ability comparing to that in control group. (Figure 4A and 4B). The results from the transwell assays also showed that downregulation of NNT-AS1 significantly impaired CRC cell migration and invasion ability in both cell lines (Figure 4C and 4D).

### Silencing NNT-AS1 attenuates EMT and MAPK/Erk signaling pathway in CRC cells

**Figure 5:**
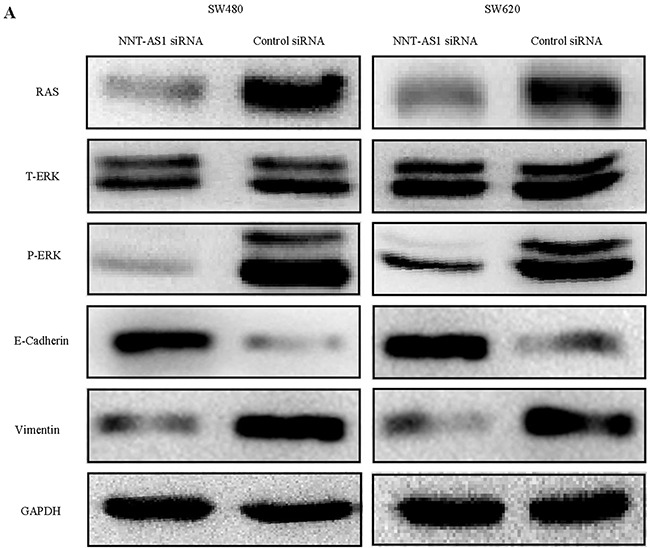
NNT-AS1 induces MAPK/Erk signaling pathway and EMT **A**. The markers of MAPK/Erk signaling : Ras, total-Erk (T-ErK), phosphorylated-Erk (p-Erk) and EMT markers (E-Cadherin, Vimentin) were analyzed by western-blot in SW480 and SW620 cell lines. GAPDH was used as a loading control.

In order to elucidate the mechanism by which NNT-AS1 regulates CRC cell proliferation, migration, and invasion, Western blot assays were performed. The results showed that Ras was significantly downregulated and Erk1/2 was less phosphorylated/activated in the cells transfected with the NNT-AS1-specfic siRNA, while total levels of Erk1/2 remained unchanged (Figure 5A). Interestingly, E-Cadherin protein expression was upregulated, and vimentin protein expression was downregulated in the cells transfected with the NNT-AS1 siRNA (Figure 5A). These results implied that NNT-AS1 regulates the activity of MAPK/Erk signaling pathway, which promotes CRC cells to undergo EMT and increases CRC cell proliferation, migration, and invasion.

### Silencing NNT-AS1 suppressed tumor growth and metastasis in vivo

**Figure 6:**
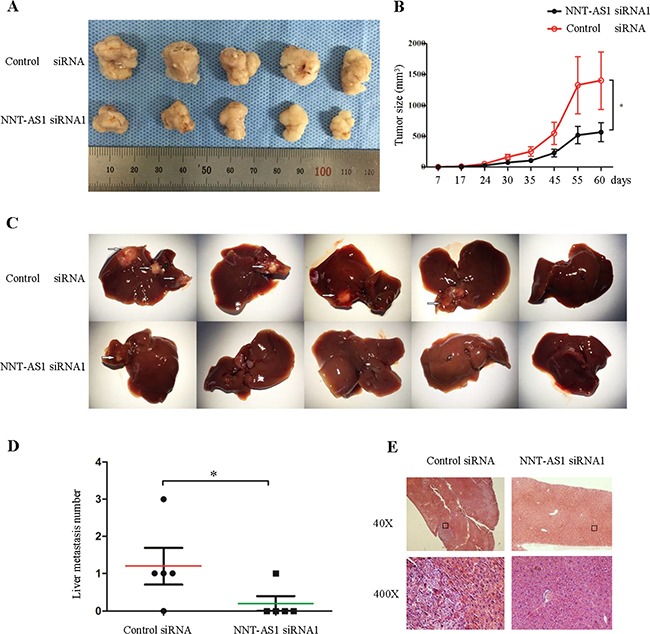
NNT-AS1 promotes CRC cell proliferation and metastasis in vivo **A**. Stable SW620 cells transfected NNT-AS1-specific siRNA vector and control vector were injected subcutaneously into five nude mice respectively. **B**. The tumor volumes were measured (*P<0.01). **C**. Liver metastasis images. **D**. Liver metastatic nodules in individual mouse (*P<0.01). **E**. The histological images of metastatic nodules in livers. Date were presented as mean±SD.

Finally, to explore whether NNT-AS1 expression affects tumor formation and growth in vivo, SW620 cells stably transfected with vectors expressing control siRNA and NNT-AS1 siRNA were subcutaneously inoculated into nude mice. All of the mice developed xenograft tumors at the subcutaneous injection sites. There was significantly less tumor mass in the NNT-AS1 knock-down group relative to the control group beginning from the 7^th^ day post-injection (Figure 6A and 6B). Furthermore, stable cells were injected into the spleens of mice to assess the metastatic potential. There were more liver metastases in the empty vector group than that in the NNT-AS1 Knockdown group (Figure 6C and 6D). Typical characteristics of tumor cells in the empty vector group by hematoxylin & eosin (H&E) staining were shown in Figure 6E. Taken together, these results demonstrate that silencing NNT-AS1 inhibits tumor growth and metastasis in vivo.

## DISCUSSION

In this study, we have shown that the expression level of NNT-AS1 in CRC cancer tissues was higher than that in adjacent noncancerous tissues. Patients with higher expression level of NNT-AS1 had shorter survival time. In CRC cell models, knocking down NNT-AS1 resulted in significantly reduced proliferation, migration, invasion in vitro and in vivo through downregulating the MAPK/Erk signaling pathway and inhibiting EMT.

Many antisense RNAs have been previously found to play vital roles in a variety of cancers. For instance, FEZ Family zinc finger 1-antisense RNA 1(FEZF1-AS1) which is upregulated in cancer tissues, has been proved to promote cell proliferation, migration and invasion in CRC [14]. ZEB1-antisense RNA 1 (ZEB1-AS1) overexpressed in hepatocellular carcinoma, is a predictor of poor prognosis [15]. Here, we identified a poorly characterized lncRNA NNT-AS1 that was upregulated in CRC tissues. Considering its higher expression in cancer tissues, NNT-AS1 can represent a useful diagnostic biomarker for CRC patients. It may also be a prognostic biomarker owing to the different survival time between patients with NNT-AS1 higher expression and lower expression and its expression positively associated with lymph node metastasis, vessel invasion in CRC patients as well as its induction of migration and invasion in cells and in nude mice.

To further investigate the functions of NNT-AS1 in CRC, we utilized siRNA-mediated knockdown of NNT-AS1 and assessed the resultant effects on cell proliferation, cell cycle, apoptosis, migration, and invasion in both SW480 and SW620 cells. We found that silencing NNT-AS1 reduced CRC cell colony formation capability. These results were further validated by our results of EdU assays, which showed that silencing NNT-AS1 significantly decreased the percentage of proliferating cells. In addition, all the results were also consistent with the results of cell cycle assays, which showed that the fraction of cells in S-phase was significantly decreased in the cells transfected with NNT-AS1-specific siRNA. Thus, NNT-AS1-mediated inhibition of cell proliferation may occur through G1 arresting. NNT-AS1 knockdown also significantly attenuated CRC cell migration and invasion based on the results of wound healing and transwell assays. What’ more, the results of the nude mice showed that NNT-AS1 promoted cell growth and liver metastasis in vivo. All these results implied that NNT-AS1 can promote cell proliferation and metastasis in vitro and in vivo.

In consideration of the above-described biological behaviors of NNT-AS1 and the relationships between NNT-AS1 and various clinicopathological characteristics, we hypothesized that NNT-AS1 may regulates important signaling pathway, such as MAPK/Erk signaling, PI3K/AKT signaling, and Wnt/β-catenin signaling. It may also associate with EMT, which is a biological process often occurring in the metastasis. Western blotting showed that NNT-AS1 knockdown impaired vimentin protein expression while upregulated E-cadherin protein expression. These results suggest that NNT-AS1 has a critical role in promoting EMT. These results also verified that MAPK/Erk signaling is activated in the presence of NNT-AS1.

During the process of EMT, E-cadherin, a cell-surface protein, decreases while vimentin, a cytoskeletal protein, increases. Studies demonstrated that EMT is associated with carcinogenesis and tumor metastasis [16]. It also demonstrated that lncRNA-mediated induction of EMT is associated with cancer metastasis. For instance, lncRNA-ATB is upregulated in hepatocellular carcinoma and promotes the invasion-metastasis cascade in this cancer by inducing EMT [17]. HOX transcript antisense RNA (HOTAIR) was proved to promote EMT in cervical cancer [18]. Metastasis associated lung adenocarcinoma transcript 1 (MALAT1) has been shown to promote cell invasion and metastasis by inducting EMT in cervical cancer [19].

The MAPK/Erk signaling pathway is one of the most important cell signaling networks in tumorigenesis. Aberrant activation of phosphorylated Erk1/2 induced by lncRNAs has been observed in most types of cancers, including glioma [20], gastric cancer [21], lung cancer [22], hepatocellular carcinoma [23], gallbladder cancer [24], and melanoma [25]. Consistent with these findings, our results implied that the effect of NNT-AS1 on CRC may rely on activating MAPK/Erk signaling pathway. But the direct link between MAPK/Erk signaling and NNT-AS1 remains unknown, awaiting further investigations.

There is a complex regulatory network between NNT-AS1 and CRC, other underlying mechanisms should be further studied. Accumulated evidence demonstrated that changing the lncRNAs expression can alter the drug resistance. For example, silencing PVT1 which overexpressed in gastric cancer cells can reduce the cisplatin resistance [26]. We can gain insight into the relationship between NNT-AS1 and drug assistance in future. Further verification using more specimens is necessary.

In conclusion, our studies demonstrated that a poorly known lncRNA NNT-AS1 plays an important role in tumor development and metastasis of CRC. NNT-AS1 is a potential diagnostic and prognostic biomarker for CRC patient.

## MATERIALS AND METHODS

### Tissue specimens and clinical data collection

This study was approved by the Ethics Review Board of Affiliated Beijing Chaoyang Hospital, Capital Medical University. Written informed consent was obtained from all patients, and the animal experiments were conducted in accordance with the Guide for the Care and Use of Laboratory Animals in Chaoyang Hospital.

70 colorectal cancer tissues and paired adjacent noncancerous tissues were collected from patients undergoing operations at the Department of General Surgery, Beijing Chao-Yang Hospital (Beijing, China), between January 2013 and May 2014.

The inclusion criteria were as follows: no preoperative chemotherapy, radiotherapy, or targeted therapy; no other types of tumors; no autoimmune diseases. The specimens obtained during surgery were immediately snap frozen in liquid nitrogen and stored at -80°C until RNA extraction. Clinical staging of the specimens was based on NCCN guidelines. After 23 to 39 months, all patients underwent telephone follow-up, and their PFS and OS rates were calculated from the time of the initial surgery.

### RNA extraction and reverse transcription

Total RNA was isolated from CRC tissues, paired adjacent noncancerous tissues and CRC cells using TRIzol reagent (Invitrogen, Carlsbad, CA, USA) following the manufacturer's instructions. RNA quality was evaluated using a Nanodrop 2000 (Wilmington, DE, USA), and RNA integrity was assessed using agarose gel electrophoresis. An aliquot of 1 μg of total RNA was reverse-transcribed into complementary DNA (cDNA) according to the manufacturer's protocol using a High-capacity cDNA Reverse Transcription kit (Applied Biosystems, Foster City, CA, USA).

### Quantitative real-time PCR

Quantitative real-time PCR (qRT-PCR) was performed using a Roche LightCycler® 480II system with TaqMan assays (Invitrogen, Carlsbad, CA, USA) (NNT-AS1 assay ID: Hs 04333390_s1; GAPDH Catalog number: 402869) and TaqMan® Universal Master Mix II without UNG (Invitrogen, Carlsbad, CA, USA) to detect NNT-AS1 expression. The transcription levels were normalized to GAPDH expression. The qRT-PCR assays were performed in triplicate reactions with the following conditions: (1) 95°C for 10 min and (2) 40 cycles of 95°C for 15 s and 60 °C for 1 min. The relative expression of NNT-AS1 was calculated using the ΔCT (Ct lncRNA-Ct GAPDH) method.

### CRC cell lines and culture conditions

The SW480 and SW620 human CRC cell lines were obtained from the American Type Culture Collection (USA). Both cell lines were maintained in L-15 Medium containing 10% FBS (Gibco, Carlsbad, CA, USA) with 100 ug/ml penicillin and 100 μg ml/streptomycin (Gibco, Carlsbad, CA, USA) and incubated at 37°C under 5% CO_2_.

### siRNA transfection of CRC cells

Silencer select small interference RNAs (siRNAs) specific to NNT-AS1 (siRNA IDs s501613 and s501614) and a control siRNA (#4390843) were obtained from Ambion (Invitrogen, Carlsbad, CA, USA). For silencing NNT-AS1 in CRC cells, the NNT-AS1-specific RNAs (5’-GAAAAGAAAAAGAAGCUUAtt-3’; 5’-GCAACA GAGUGAUACUCUAtt-3’) and control siRNA (5’- TTCTCCGAACGTGTCACGT-3’) were transfected into SW480 and SW620 cells using Lipofectamine RNAiMAX (Invitrogen, Carlsbad, CA, USA) and Opti-MEM (Gibco, Carlsbad, CA, USA) according to the manufacturer's recommendations.

### Stable cell line generation

The NNT-AS1-specific siRNA sequence 5’-GAAAAGAAAAAGAAGCTTA-3’ and the control siRNA sequence 5’- TTCTCCGAACGTGTCACGT-3’ were separately cloned into the GV102 vector by GeneChem (shanghai, China). SW480 and SW620 cells were transfected with the empty vector or the siNNT-AS1 vector using Lipofectamine 3000 (Invitrogen, Carlsbad, CA, USA) according to the manufacturer's protocols. Single colonies were selected using G418 (0.5 mg/mL), and then the stable SW480 and SW620 cell lines were generated.

### Soft agar colony formation assays

SW480 and SW620 cells, which were stably transfected with vectors, were submitted to soft agar colony formation assays in six-well plates. After the cells were trypsinized, each well was filled with 1 ml 0.7% agar solution with L-15 media containing 10% FBS and 2% penicillin-streptomycin containing 6×10^2^ cells on the growth layer, which was layered on top of a 1.2% agar layer. The plates were incubated for 2 weeks at 37°C under 5% CO_2_, and the resultant colonies were counted using ImageJ. All measurements were repeated three times in triplicate.

### Cell proliferation assays

After transfection for seventy-two hours, 20 μL ClickREdU solution (Invitrogen, Carlsbad, CA, USA) was added to the culture medium at a concentration of 10 μM in six-well plate and mixed well. During this process, proliferating cells were labeled with EdU. As a negative staining control, cells from the same population that were not treated with EdU solution were used. Subsequently, the cells were harvested after 2 hours, fixed and permeabilized according to the manufacturer's instruction. Then, EdU staining was analyzed by flow cytometry using a BD FACSCanto II (BD Biosciences, San Jose, CA) and evaluated using BD FACSDiva software. All measurements were repeated three times in triplicate.

### Cell cycle analysis

CRC cells were transfected with NNT-AS1 siRNAs or Control siRNA. After transfection for seventy-two hours, 1×10^6^ cells were harvested by trypsinization and fixed with 10 ml cold 70% ethanol overnight. Subsequently, the cells were washed in PBS and treated with 500 μl FxCycle^TM^ PI/RNase Staining Solution (Invitrogen, Carlsbad, CA, USA) containing DNase-free RNase A and a permeabilization reagent for 30 min at 37°C while protected from light. The percentages of cells in various phases of the cell cycle were determined using a BD FACS Canto II (BD Biosciences, San Jose, CA) and analyzed with BD FACSDiva software. All measurements were repeated three times in triplicate.

### Cell apoptosis analysis

CRC cells were transfected with NNT-AS1 siRNAs or Control siRNA. After transfection for seventy-two hours, the cells were harvested and washed in cold PBS. The cells were evaluated using the manufacturer's protocol included in the Pacific Blue^TM^ Annexin V/SYTOXR AADvanced^TM^ Apoptosis Kit (Invitrogen, Carlsbad, CA, USA). Briefly, the supernatant was discarded, and the cells were resuspended in 1X annexin binding buffer at a concentration of 1×10^6^ cells/mL to a volume of 100 μL per assay. Then, 5 μL of Pacific Blue^TM^ annexin V and 1 μL of 500 Um SYTOX AADvancedTM Dead Cell Stain working solution were added to each 100-μL aliquot of cells. The cells were incubated at room temperature for 30 minutes protected from light. After the incubation period, 400 μL of 1X annexin binding buffer was added to the cells and mixed gently, and the samples were kept on ice. As soon as possible, the stained cells were analyzed by flow cytometry. The fluorescence emission was measured using a 450 nm bandpass filter (equivalent to excitation with 405 nm)(Pacific Blue^TM^ dye) and a 670 bandpass filter (equivalent to excitation with 488 nm)(SYTOX AADvanced). All measurements were repeated three times in triplicate.

### Wound healing assay

CRC cells were transfected with NNT-AS1 siRNAs or Control siRNA. Seventy-two hours after transfection, the cells were harvested and seeded into 6-well plates. After the cells reached approximately 70% confluence, a wound was created using a pipette tip. The wells were then washed, and serum-free medium was added. The cells were photographed every 12 h for 2 days to measure wound healing. All measurements were repeated three times in triplicate.

### Cell migration and invasion assays

CRC cells were transfected with NNT-AS1 siRNAs or Control siRNA. Seventy-two hours after transfection, 2×10^4^ cells were harvested and placed in 100 μl serum-free medium in the top chamber of a well coated with Matrigel (BD Biosciences, San Jose, CA, USA). Complete medium containing 10% FBS was added to the bottom chamber. After 24h (migration) and 50 h (invasion) incubation at 37°C under 5% CO_2_, the cells that had migrated through the Matrigel were fixed with methanol for 30 min and then stained with crystal violet for 15 min. The number of invaded cells was quantified using ImageJ.

### Western blot assay and antibodies

CRC cells (SW480 and SW620) were transfected with NNT-AS1 siRNAs or Control siRNA in 6-well plates. Seventy-two hours after transfection, the cells were washed twice with cold PBS, and 30 μL of RIPA (Solarbio, Shanghai, China) containing a protease inhibitor cocktail (Invitrogen, Carlsbad, CA, USA), a phosphatase inhibitor cocktail (Invitrogen, Carlsbad, CA, USA) and 2 mM ethylenediaminetetraacetic acid (EDTA) at pH 8.0 was added to each well. Following this, the cells were collected into a cold tube. Cell lysates were centrifuged at 12,000 g for 20 min at 4°C. Subsequently, the proteins in the lysates were separated on a 10% polyacrylamide gel and transferred onto polyvinylidene difluoride (PVDF) membranes. The membranes were blocked with 10% non-fat milk for 4 h at room temperature and then incubated with primary antibodies for 1 h at 4°C. Antibodies against the following proteins were used: E-cadherin (24E10), vimentin (D21H3), p44/42 MAPK (Erk1/2), phospho-p44/42 MAPK (Erk1/2) (Thr202/Tyr204) (D13.14.4E), GAPDH (D16H11) (from Cell Signaling Technology, Danvers, MA) and Ras [EP1125Y] (from Abcam, Cambridge, UK), followed by second antibody (zhongshanjinqiao, China). Protein bands were visualized using Super Enhanced Chemiluminescence Detection regents (Applygen Technologies, Beijing, China).

### Xenograft assay in nude mice

Five-week-old male nude BALB/c mice were purchased from Charles River Laboratories (Beijing, China) and acclimated for one week under standard conditions at the Medical Research Center of Beijing Chaoyang Hospital prior to experimentation. The animals were maintained according to the Guide for the Care and Use of Laboratory Animals. SW620 cells, which were stably transfected, at a concentration of 1×106 were then subcutaneously injected into the right flank of each nude mouse (5 vs. 5), and tumor volumes were measured every several days for 60 days. In addition, we created a liver tumor metastasis model by injection of stably transfected cells into the spleens of the mice (5 vs. 5). HE analysis was employed.

### Statistical analyses

All statistical analyses were performed using SPSS version 18.0 (SPSS, Chicago, IL, USA) and GraphPad Prism 5.0 (GraphPad software, La Jolla, CA, USA). All measurements were repeated three times in triplicate. Significant differences between NNT-AS1 expression levels in cancer tissues and noncancerous tissues were determined using the Wilcoxon matched pairs test, and the clinicopathological features of the different subgroups of CRC patients were evaluated using chi-square tests. The univariate Kaplan-Meier method and the multivariate Cox regression model were used for survival analysis. P values less than 0.05 were considered statistically significant.

## Abbreviations

LncRNA: Long noncoding RNA
NNT-AS1: Nicotinamide Nucleotide Transhydro-genase-antisense RNA1
MAPK: Mitogen activated protein kinase
Erk: Extracellular regulated MAP kinase
EMT: Epithelial-mesenchymal transition
CRC: Colorectal cancer
HOTAIR: Hox transcript antisense intergenic RNA
XIST: X inactive specific transcript (XIST)
CCAT2: Colon cancer associated transcript 2
ORF: Open Reading Frames
CPC: Coding Potential Calculator
PFS: Progression-free survival
OS: Overall survival
